# The depressive situation

**DOI:** 10.3389/fpsyg.2013.00429

**Published:** 2013-07-17

**Authors:** Kerrin A. Jacobs

**Affiliations:** Kolleg Friedrich Nietzsche, Klassik Stiftung WeimarWeimar, Germany

**Keywords:** depression, *existential situation*, experiential synthesis, *practical sense*, habitus, narrativity, evaluative coherence, caring

## Abstract

From a naturalistic perspective on mental illness, depression is often described in terms of biological dysfunctions, while a normative perspective emphasizes the lived experience of depression as a harmful condition. The paper relates a conceptual analysis of “depressive situation” to an analysis of the lived experience of depression. As such, it predominantly aims to specify depression as a harmful condition in lights of normative perspective on mental disorder, but partially refers to empirical research, i.e., naturalistic perspective on depression, to exemplarily stress on the methodological merits and limits of relating phenomenological considerations closer to empirical research. The *depressive situation* is further specified with an examination of the evaluative dynamics by which individuals meaningfully relate to themselves, others and the world. These evaluative dynamics emerge out of the interplay of pre-reflective and reflective processes, which are significantly altered in depression. Such alterations of the evaluative structure are inextricably intertwined with significant distortions of practical sense in depression. From a phenomenological perspective, these distortions of practical sense show in characteristic experiences of evaluative incoherence and impairments of agency. Finally, this paper focuses on an examination of “evaluative incapacity,” which has the integrative potential to capture a range of typical changes of meaningful relatedness that determine the depressive situation.

## INTRODUCTION

The paper aims to concretize some phenomena involved in depression, according to which it is conceptualized as harmful condition from a normative perspective. Rather than positioning my analysis of the depressive situation in fundamental opposition to the value-neutral perspective on depression provided by naturalism, it rather affirms the value of both perspectives from a methodological perspective. Although a conceptual analysis and phenomenological considerations of the depressive situation clearly face some methodological limitations in accounting for the empirical research on depression, this approach might indicate an alternative view on depression that can also inform naturalistic accounts. The lived experience of depression is often neglected in the descriptions of depression in terms of biological dysfunctions (cf. Jacobs and Walter, 2011). This can be counterbalanced in relating empirical research on depression closer to phenomenological considerations, for instance, in relating an analysis of particular depressive experience to those theories that aim to provide the neuropsychological correlates to it. Inasmuch as my analysis of changes in the evaluative experience of self and world in depression is exemplarily substantiated by autobiographic narratives, this illustrates, in which ways depressive experience differs in many respects from non-depressive encounters with the world. From a phenomenological perspective on depression, these narratives are authentic expressions of the lived experience of depression and for this reason also provide an additional diagnostic value for clinical diagnosis. The reported phenomena in depression narratives point beyond the well-known clinical cluster of classificatory criteria for depression. The *Diagnostic and Statistical Manual of Mental Disorders, 4th Edition*, Text Revision (*DSM-IV-TR*; American Psychiatric Association, 2000) and *International Classification of Diseases, Tenth Revision* (*ICD-10*; WHO, 2010) criteria of depression often do not reflect the experiential heterogeneity, as well as the subtle differences in the experience of depression. Such phenomena, which are neither captured by the clinical diagnostic manuals, nor become transparent as lived experiences from a naturalistic perspective, are, for instance, significant changes in one’s sense of reality, alterations in the experience of one’s abilities (changes of practical sense), as well as a range of other experiential alterations in one’s sense of meaningful relatedness to the world. Consequently, my analysis aims to enrich the clinical picture of depression drawn by the *DSM* or *ICD* with respect to a more detailed analysis of the underlying evaluative dynamics that shape the depressive situation. In doing so it may contribute to a reconciliation of the scientific and everyday characterization of depressive symptoms, and may show how a normative understanding of depression as *illness* completes a naturalistic conceptualization of depression as *disease*^1^.

Before explaining the particular evaluative dynamics that underlie these experiences in depression in greater detail, one can start with some conceptual considerations on the term “existential situation,” which addresses those processes in which individuals situate themselves in the world in a particular evaluative way. But how are individuals situated by the ongoing dynamics of evaluative self- and world-disclosure that emerge out of the general interaction between organism and world? It is assumed that these dynamics are the structural prerequisite placing individuals already in some sort of *meaningful* relation to the world. This bond of meaningful relatedness, this structure, which is instantiated by and maintained in proceeding evaluative experience and practice, points to the inherently evaluative dimension of self- and world-disclosure, thus, to the normative depths of existential situatedness.

With the concept of “depressive situation,” I allude to what Karl Jaspers has coined *border situation* (German: *Grenzsituation*), a notion which can be used to describe psychiatric disorders as an exceptional state of existence, *converting* “situations of daily life” (German: *Alltagssituationen*) to border situations, inasmuch as fundamental alterations of evaluative processes of self- and world-disclosure are involved (cf. Jaspers, 1925, 1973; Jacobs and Thome, 2003; Fuchs, 2008). The concept of *evaluative self- and world-disclosure* (German: *Selbst- und Welt-Erschließung*) allows us to refer to those evaluative processes in which things in the world, other people and also (aspects of) oneself and one’s actions become intelligible to someone by virtue of being part of a world that provides a background of meaning to one’s (practical) encounters (cf. Kompridis, 2006). The main hypothesis of this paper is, that the existential situation of the depressive type – the depressive situation – is characterized by such structural changes in evaluative processes, which contribute to characteristic experiences and modes of enaction that differ significantly from non-depressive ones. These dynamics of self- and world-disclosing significantly alter in depression, i.e., what appears as intelligible to one often dramatically changes in depression: depressed persons often report that they feel disconnected from the world, that it appears as an empty place deprived of all meaning, that other people and activities formerly enjoyed are no longer of interest, that they get stuck in deliberative processes of rumination and indecisiveness, etc. Inasmuch as the sheer variety of experiences symptomatic of depression cannot become fully addressed here, I predominantly focus on conceptual considerations about evaluative self- and world-disclosure. These may prepare the grounds for testing, which particular experiences and clinical symptoms point to an inherently evaluative problematic of the depressive situation.

## EXISTENTIAL SITUATION – THE DYNAMICS OF EVALUATIVE SELF- AND WORLD-DISCLOSURE

For a start, it has to be mentioned that in contrast to approaches suggesting *affective* intentionality as the conceptual core of meaningful self-world-relatedness (Stephan and Slaby, 2011), or which focus predominantly on *feelings* as providing this evaluative structure for modes of relatedness (Ratcliffe, 2008), I argue for a *broader notion of intentionality*, which can account for a greater variety of evaluative modes that structure one’s existential situation in general, and by which in particular he heterogeneity of changes in depressive evaluation, and not just particular changes of affect, is addressed. It is assumed that processes of evaluative self- and world-disclosure cannot be reduced to or equated with a mere affective (or: “felt”) dimension, albeit affectivity (e.g., experiencing an emotional episode) certainly has a self- and world-disclosing function and, as such, provides one way to address a certain mode of evaluative self-world-relation, respectively (cf. Jacobs, 2013a).

To argue for a broader notion of intentionality then also implies to consider the whole *bodily corporal* dimension as irremissibly constitutive of particular modes of meaningful relatedness (cf. e.g., Ratcliffe, 2009; Fuchs et al., 2010). This likewise does not imply to restrict the self- and world-disclosing function of the lived body to a pure feeling dimension in terms of affectivity. Evaluative self- and world-disclosure through the lived body neither depletes in affective states, nor does it solely rest on *felt* evaluations (cf. Helm, 2002). Rather the dynamic interplay of these with different types of incorporated social structures (e.g., values) and particular “knowledge” (practical skills, unconscious desires, embodied memories, etc.) has to be reconsidered as significantly contributing to the respective modes of evaluative relatedness provided by the bodily corporal dimension.

Generally, the body as *Körper* expresses several forms of incorporated practice or roles, e.g., an actor can, for instance, incorporate (*verkörpern*) a certain role. While this kind of incorporation is rather the product of intentional processes (e.g., learning to incorporate a role), there are also specific forms of incorporation that “enter” the body through social structures, education, etc., which rather tacitly shape our situation as embodied individuals. Contrastingly, the *Leib* is the lived body, the transparent medium for world-disclosure that enables us to engage with the world (cf. Jäger, 2004). Pierre Bourdieu’s theory of embodied practice paradigmatically outlines how external (social, cultural, moral) structures become incorporated in a literal sense. With his notion of *habitus*, he has elaborated individual (pre-)dispositions in terms of an embodied, embedded and enacted perspective on individual situatedness (cf. Bourdieu, 1979, 1980). This reading of *embodied practice* is the presupposed rationale for my analysis of the evaluative dynamics of psychopathological self- and world-disclosure (cf. Jacobs, 2012, p. 251, Jacobs).

Inasmuch as it is rather the complex *interplay of different types of evaluations and evaluative states* – which together contribute to how someone actually experiences a specific situation, and is directed toward the world and others in a meaningful way – this interplay can be further explained by the procedural dynamics of evaluative processes. These considerations provide the prerequisites for the analysis of how these procedural dynamics of evaluative processes change in depression, and how this, in return, contributes to characteristic changes of meaningful relatedness.

### PRE-REFLECTIVE AND REFLECTIVE PROCESSES

Generally, the procedural dynamics of evaluative self- and world-disclosure can be specified in pointing to the particular interplay of the pre-reflective and reflective sphere of one’s existential situation (cf. Jacobs, 2012, 202ff). The pre-reflective sphere is – broadly construed – that according to which all intentional encounters with the world are pre-structured by a set of individual predispositions (for instance perceptional schemes, embodied skills, tastes, capabilities, etc.). It has its counterpart in a sphere of reflection, while it is particularly by self-reflexive processes^2^ that individuals structure their life as autonomous agents (cf. Jacobs, 2012, 175ff; pp. 221–229; 242ff).

These modes of self-directedness in acts of reflection, of course, form a special kind of evaluative self-relation, which differs from that provided by the pre-reflective sphere: inasmuch as individuals are able to develop an objective *stance* (German: *Haltung*) toward their own beliefs, desires, feelings, behaviors and actions, it is in virtue of their self-reflexive capacity that they *situate themselves as autonomous agents* in fields of social (e.g., moral, cultural, etc.) practice (cf. Rothacker, 1942, 55ff; Jaspers, 1973, 203). In contrast to theories of practice, in which the self-reflexive stance and associated rational strategies of self-constitution, for instance, in deliberative processes play rather a secondary role (e.g., Bourdieu, 1979), one can emphasize the experiential and practical modes of meaningful relatedness provided by these. This implies that a person can influence by which particular evaluative stance, and by which particular modes of practice, she relates to herself, others and the world. These modes of being directed to the world (and others) then have their vital counterpart in the evaluative modes of self- and world-disclosure provided by the pre-reflective sphere. Both spheres equally contribute to how one’s existential situation enfolds in the procedural dynamics of evaluation. Consequently, the depressive situation can be explained by focusing on structural changes of evaluative processes that take place on the pre-reflexive and reflective/self-reflexive level.

### THE INTERPLAY OF DIFFERENT TYPES OF EVALUATION

If one aims to account for the *interplay of different types of evaluations* that equally shape particular evaluative experiences and modes of meaningful relatedness, and if one further stresses the complex evaluative phenomenality and intentionality provided by the dynamics of both spheres, one has to differentiate at least those types of evaluations that rather refer to a pre-reflective sphere, from those that predominantly structure the more reflective encounters with the world: the pre-reflective sphere provides one with an experiential structure and particular modes for self-world-relation that can be described as stemming from rather spontaneous and immediate evaluations (e.g., in terms of bodily appraisals). Often this pre-reflective sphere is described as an evaluative dimension, which rather tacitly structures one’s daily encounters with the world. This evaluative “rather taken for granted-structure” has been specified, for instance, in terms of basic existential feelings (cf. Ratcliffe, 2008; Jacobs et al., 2013). These background orientations provide one with a basic sense of belonging or a sense of reality, thus, with an evaluative structure that normally is rather the unquestioned evaluative basis for the more reflective processes of self- and world-disclosure. They come to the focus of someone’s attention especially when they significantly alter, e.g., due to a slight experiential fracture of one’s sense of reality as in a *déjà* vu-experience. Other classes of evaluative types that rather seem to belong to the pre-reflective realm are, for instance, incorporated memories and unconscious desires.

What unites all these different types of pre-reflective evaluations is that they have to be reconsidered for their constitutive role for agency, i.e., as (partially) constitutive of self-reflexive evaluative processes. The discrimination of a pre-reflective vs. reflective evaluative sphere of one’s existential situation thus is a purely analytical one, as this paper emphasizes the *dynamic interplay of both spheres* that establish the evaluative processes – the evaluative structure – by which the meaningful bonds between self and world emerge. Claiming a primacy of self-reflexive evaluative processes for (e.g., moral) self-constitution as agent does not imply to dismiss that both classes of evaluations contribute to such structurally complex modes of evaluative directedness.

## THE SYNTHESIS-VIEW – THE INTEGRATIVE UNITY OF EVALUATIVE EXPERIENCE

It may have become transparent that it is by experiencing a particular type of evaluative state (e.g., a desire) that we are already directed to the world and, as such, imbue the world with meaning. Consequently, the intentional and the phenomenal dimension are no longer separable in evaluative processes, but rather must be conceptualized as essentially *unified in the evaluative experiences of self and world*.

From what has been said so far follows that the particular evaluative modes of existential situatedness can be characterized *neither* solely in terms of mental states (mere intentionalism), as the concept of intentionality has been broadened by considering the whole bodily corporal dimension of individuals as something essential to the respective (evaluative) experience itself. *Nor* are the evaluative modes by which the self-world-relation is instantiated are understood as just being comprised of separable *components*, whose relational connection often raises conceptual problems. From the perspective of such component theories, evaluative states (e.g., emotions) are described as having a certain kind of intentionality (world-to-mind/mind-to-world/direction of fit/or both, in being “*janus-faced*” cf. de Sousa, 2002) *plus* an other component to account for the phenomenological dimension of evaluative states. Whatever might be considered to be a defining factor of a certain type of evaluation seems to be simply *added on* as a further component to its intentional content.

One might object that my account faces this common problem, in failing to provide the required sufficient explanation of the interrelation of the components by which a particular type of evaluative state (for instance, an emotion) must be comprised. And inasmuch as different types of evaluations amount to the more complex evaluative meta-structure of one’s actual existential situation, one has to present a theory that equally can account for the structural connectivity of evaluative content, too.

This can be tackled the following way: rather than focusing on an analysis of how these “components” either combine to constitute a particular evaluative state, or how different evaluative types exactly must be combined to form more complex evaluative patterns, these factors must not be treated as disjointed components: neither for the case of a particular evaluative state, nor for the case of a complex evaluative pattern, which structurally integrates the phenomenality and intentionality of more than one evaluative type in the process of evaluative experience. One can rather aim to explain, how these elements are necessarily interrelated due to a *synthesis of evaluative experience*. This “synthesis-view” aims to take the phenomenology and intentionality as an integrative unity in evaluative experience fully into account^3^. Then the various “aspects” of being, for instance, in a particular type of evaluative state are perceived rather as *dimensions of the unifying structure of evaluative experience itself.* If one expands the synthesis-view, one can address the structural complexity of someone’s actual evaluative situation *as a whole*: the different types of pre-reflective and reflective evaluation thereby are perceived as building the structure of more complex evaluative patterns by which several types of evaluative content become integrated. This means that different types of evaluations (e.g., desire, emotion, or the normative evaluation in case of belief, etc.) become structurally related with each other to form a more complex evaluative pattern for evaluative self- and world-disclosure. With this, one can conceptually address the evaluative totality, and, simultaneously, the procedural dynamics of evaluative self- and world-disclosure.

## NARRATIVITY AS THE STRUCTURING PRINCIPLE OF EVALUATIVE EXPERIENCE

But one may still ask how such a *structuring principle* that guarantees for the experiential unity in processes of evaluative self- and world-disclosure may be described. This has to be explained in more detail, as I argue in the following steps that many forms of depressive experience can be explained due to fragmentations of this experiential unity.

### THE NARRATIVE STRUCTURE OF EVALUATIVE EXPERIENCE

This is exemplarily outlined in Voss (2004, 185ff) thoughtful narrative account, in which she elegantly solves the component problem for the particular subclass of evaluative states, namely the emotions. In her analysis, *evaluative narratives* are carefully distinguished from *narration*. While “narration” is the result of purposive writing and speech, the evaluative narrative is the meaningful structure, which can become the *object of a narration*. Insofar as narration is an appropriate medium for transporting evaluative content, one can argue, in line with her account, for the *narrative structure of evaluative experience* in general. This plays a constitutive role for uniting phenomenality and intentionality in evaluative experience: narration is then the appropriate or inappropriate description of a desire, belief, (bodily) feeling, emotion, etc., which already points to the narrative structure of the particular evaluative content of a specific evaluation. More precisely: it points to the evaluative narrative as the *situational instantiation of the formal object of a certain kind of evaluative type*. The proposition of an evaluative content is never isolated from a corresponding, more detailed narrative structure of a specific evaluative state. Accordingly, the idea of a narrative structure of evaluative states suggests, that one therein refers to the specific evaluative content and structure of these states. As such, one refers to certain types *of significance or import*, which are instantiated in a specific situation. Thus, experiencing a particular type of evaluative state is equivalent to experiencing significance, to which a particular valence is always integral. This valence dimension has been exemplarily discussed with regard to the emotions, in particular, for its motivating role (e.g., Helm, 2010, 2001, 99ff). These experiences – that something or someone is experienced as having import, is of significance or is experienced as meaningful – are not solely provided by the reflective sphere, e.g., in virtue of self-reflexive processes (e.g., in terms of “caring,” cf. Frankfurt, 1988; Jacobs, 2012, pp. 219–228), but also by the pre-reflective sphere, e.g., in terms of bodily appraisals (cf. Prinz, 2004, p. 77, 173) and other bodily corporal processes that positively or negatively reinforce individual practice of meaningful relatedness.

### THE EXPERIENTIAL UNITY

The constitution of a* phenomenal unity* and its respective situational occurrence in the experience of a particular evaluative states then can be explained the following way: certain types of evaluative states are linked with evaluative concepts and their respectively embedded propositional content, which form the underlying “core”-intentional structure of an particular evaluative state; while the whole narration (e.g., in form of an oral or written report) conveys the structure of that evaluative content. The “evaluative narratives” are the more fine-grained structures, which contribute to the narrative *coherence* of the more “robust,” single evaluative concepts of evaluative experience (cf. Slaby, 2008, p. 274). Such evaluative concepts (like, for instance, “threatening,” “dangerous,” etc.) that relate to the formal object (e.g., psychopath) of the respective evaluative type (e.g., anxiety) thereby amount to the *all-encompassing conceptual structure of a particular evaluative state* (e.g., of an emotion), which is expressed (e.g., communicated) in narration. Consequently, one can also account for the structural connectivity *of different evaluative states* in complex evaluative situations by pointing to *narrativity* as an adequate structuring principle. Then the complex phenomenality and intentionality of different types of evaluations become integrated into the *all-encompassing conceptual meta-structure of one’s evaluative situation as a whole*. This refers to a synthesis according to which different experiential modes of evaluation become structurally interrelated in complex evaluative experience. Experiencing, e.g., anxiety when standing face to face with a psychopath then becomes structurally connected with isochronally occurring evaluative experience, for instance, with the desire to run away, the belief that one has to protect the child one hides behind one’s back, with bodily feelings of being close to collapsing, etc., according to which someone’s actual evaluative situation enfolds. This strengthens the hypothesis of a procedural dynamics of evaluation, i.e., it points to the continuous processes by which the evaluative structure of one’s existential situation develops and by which one experientially traverses one’s actual existential situation in multifaceted modes of meaningful relatedness.

### THE NARRATIVE PARADIGM AND DEPRESSION

Although there are alternative ways to conceptualize the unity of evaluative experience, and albeit I do not claim narrativity to be a necessary condition for the conceptualization of the experiential synthesis in evaluative experience, it presents as a quite reasonable paradigm; especially, if one aims to explain the (structural) changes in depressive evaluation. One can describe on a conceptual level, how particular experiences of *evaluative incoherence* can be traced back to *inconsistencies* (typical deformations) in the evaluative structure itself. These cause “fragmentations” of the experiential unity in processes of evaluative self- and world-disclosure in depression^4^. Very often, such fragmentations of the experiential unity are already reflected in the incoherent styles of writing and speech in depressed patients (cf. Hilken, 1993; Hunsaker Hawkins, 1999). As such, *pathography* (Möbius, 1907) provides a very good insight to recurrent and significantly altered evaluative patterns of experiences in psychopathology (cf. Habermas, 2011). Consequently, I refer to narratives of depression to exemplify such experiences of evaluative incoherence. These may put some flesh to the conceptual bone of “depressive situatedness.”

Moreover, narration is a particular type of sense-making-practice, in which evaluative content (experiences of import) is transformed – e.g., by verbal expression – to a speech *act*, and, as such, provides one way to address how evaluative experience instantiates and expresses in individual meaningful practice. The important role it plays in processes of coping, identity formation (cf. Bruner, 2001; Habermas, 2012) and self-knowledge (cf. Bruner, 1987) points also to a therapeutic value of the narrative paradigm (cf. Richert, 2006). This already has been pointed out by Freud (1905, p. 7) who characterizes neuroses as “gaps” in autobiographic narratives.

Given the plausibility of such a holistic, dynamical perspective on the individual evaluative setting, my analysis affirms a synthesis-view as essential for a description of experiential and practical changes of meaningful relatedness in depression.

## THE DEPRESSIVE SITUATION – CHANGES OF PRACTICAL SENSE AND MEANINGFUL RELATEDNESS

It is next outlined what characterizes the *depressive situation*. I suggest that those processes in which different types of evaluation/evaluative states normally become structurally interrelated and integrated to consistent evaluative patterns for self- and world-disclosure are more prone to distortion in depressive as in healthy individuals. I focus in the following on how the otherwise “smooth” and flexible procedural dynamics of evaluation – denoting the openness of one’s existential situation – come unstuck and therein amount to symptomatic depressive experiences of changes of meaningful relatedness, i.e., to particular experiences of evaluative incoherence and incapacity. These are addressed as distortions of practical sense in depression.

### EXISTENTIAL OPENNESS AND ITS RESTRICTION IN DEPRESSION

Inasmuch as we situate ourselves in the world in ongoing processes of evaluative self- and world-disclosure, our existential situation normally *remains receptive* for experiential changes and different modes for meaningful relatedness, respectively. In contrast, the depressive situation rather points to the opposite of an existential openness.

Generally, one’s meaningful self-world-relation evolves out of the proceeding dynamics of the pre-reflective and reflective evaluative spheres that provide the evaluative architecture of the existential situation. With these procedural dynamics one can, for instance, account for the coming and going of different evaluative states in a specific situation, as well as for the individual variances in evaluative reactions to the very same event, i.e., how individuals flexibly adapt to contextual constraints in a specific situation. In the continuous processes of registering, adopting, maintaining, reflecting or rejecting certain beliefs, desires, feelings, fantasies, values, ideals, etc. one navigates through the world as an evaluative being in more or less meaningful ways.

#### Habitual attunements

It has to be mentioned that a set of *relatively stable* (not rigid!) evaluative patterns for self-understanding and world-orientation become individually acquired. These evaluative patterns form a *habitual evaluative repertoire* that narrow a total existential openness, as it terminates the individual space of evaluative possibilities to a realm of actual evaluative capacity an individual has in specific situations. It naturally determines, for instance, the range of actions that might count as reasonable to one in a specific situation, and someone’s evaluative responsiveness to a certain event. As such, these evaluative patterns already provide a (pre-)normative basis according to which we come to appraise (pre-)reflectively what makes sense to us or what is of importance in specific contexts of social interaction. This evaluative repertoire can also become the object of reflection, for instance, in processes of contemplation about the values, ideals, concerns, etc. that often deeply impregnate one’s self-understanding and world-orientation.

Inasmuch as the existential situation in principle remains receptive to the changes of the evaluative structure, an essential structural requirement is provided for not experiencing oneself as fundamentally exposed to these processes. We normally experience ourselves not as fully determined in thought, feeling, desire, and by our bodily (pre-)dispositions, but rather as autonomous, thus, responsible agents (cf. Jacobs, 2012, 2013a). In contrast, the *depressive situation* is often shaped by the experiences of being existentially exposed, particularly, when depressive individuals register how their meaningful relation to the world, to others and their self-understanding has significantly changed. Such a decrease of existential openness exhibits, for instance, in a generally reduced sense for one’s actual possibilities in life, e.g., due to a changed outlook on the world filtered by negative bias. It reveals in experiences of evaluative incoherence, for instance, in moments of being torn between conflicting desires, emotions, and in particular experiences of failures of intentional action (i.e., specific *inabilities*), or problematic coping strategies for dealing with such experiences of “losing grip” on one’s life.

#### Depressive situation and (evaluative) incapacity

It is generally useful to differentiate between different realms of (in)capacity for a systematization of specific types of impairments involved in psychopathology, while the concept of “evaluative incapacity” is special for its integrative potential (Jacobs, 2012, 141f). One can argue for conceptual primacy of evaluation/evaluative incapacity in depression, insofar as specific affective/emotional, conative/volitional, and cognitive/rational disturbances in depression either present as particular subtypes of evaluative incapacity and/or significantly contribute to it. This mirrors the conceptual claim that the significant changes in the evaluative structure in depression, neither can perceived as exclusively based on irrationality (it is not just a matter of false beliefs), nor exclusively stemming from an emotional-affective, or predominantly volitive, or mere bodily corporal dimension. Insofar as different kinds of evaluations become interrelated and shape the special evaluative architecture of the actual depressive situation, one can account for different kinds of inabilities that specify the dimensions of evaluative incapacity in depression.

A prominent way to address from a naturalistic perspective on mental disorder what underlies such experiential and practical changes in depression is to refer to altered information processing in depression. One can exemplarily stress on cognitive *bias*^5^ (e.g., cf. Beck, 1963, 1987; Clark et al., 1999) in reasoning and in processing of emotional information, including attention and memory (e.g., cf. MacLeod et al., 1986; Williams et al., 1997; Gotlib and Neubauer, 2000; Gotlib et al., 2004; Joormann and Gotlib, 2007), and *distortions* in logical thinking (e.g., Ellis, 1962) in depressed individuals. These count either as vulnerability factors, or as already symptomatic for the depressive situation. These descriptions of involved dysfunctional processes provided by clinical research on depression can be related more closely to the phenomenological analysis of depressive experience, insofar as these refer to the neuropsychological correlates. In the following section, I address those as “pathogenic restrictions of existential openness” in depression. These restrictions appear more specific in lights of these biases, for instance, the “rigidity” of certain evaluative patterns for self- and world-disclosure.

#### Depressive bias and rigid evaluative patterns

This rigidity can be explained in terms of negative biases which have to be considered for both, their role in implementing the rigidity on a structural level of evaluation, and for simultaneously representing it. Depressed individuals show a tendency to focus explicitly and exclusively on their own alleged negative traits, inabilities and failures. These biases already represent how certain affects, beliefs and desires that formerly might have stood rather in contrast to one’s evaluative patterns for self- and world-disclosure and self-understanding, deeply infiltrate a persons’ evaluative system and install a different evaluative dynamics. As such, biases represent and contribute to a reduction of the existential situation to only a fractural amount of experiential and practical possibilities for meaningful relatedness, as there have been before the onset of depression. Many of the evaluative patterns that have provided one with possible and actual modes for evaluative self- and world-disclosure so far lose their practical significance, because other evaluative pattern stake over the depressive person’s life.

It has become transparent that not only specific* feelings*, but also characteristic *beliefs*, i.e., their normative evaluative content (“I am a terrible, ugly, selfish, unworthy, etc. person and deserve to suffer”) and *desires* (“I just want to die”) together with the evaluative bodily corporal rationale (e.g., psychomotor agitation, pain, loss of appetite, etc. cf. DSM-IV-TR (American Psychiatric Association, 2000), are considered characteristic types of evaluation that shape the self- and world-relation of the depressed. Being in such a way attuned to the world, depressed persons find themselves evidenced by very single negative experience. These confirm the structure of evaluation represented by the biased view. Such experiences, and respective appraisals, loop back, i.e., become re-incorporated, to the very evaluative structure from which they have arisen. The development and manifestation of the depressive self- and world-relation follow the logic of self-priming looping dynamics: the more one experiences and behaves in a “depressed” way, the more these experiences become woven into one’s personality structure, which then contributes simultaneously to the (re-)production of certain behavioral patterns. It is this kind of vicious circle by which the depressive situation becomes manifest in rigid patterns for world- and self-disclosure.

Besides vulnerability factors and individual difference in one’s habitual (pre-)dispositions, of course, specific experiences, e.g., such of reinforcement by the environment and one’s own individual practice of dealing with these experiences influence these self-priming dynamics. In order to alter both, incorporation and enacting of these structures, something from the “outside” has to come (e.g., therapeutic invention) or from the “inside” (e.g., self-reflexivity or introspection) that may eventually re-shape these perpetuating dynamics. Consequently, it is an aim of therapy to “crack” the structural rigidity in order to alter the experiential and practical modes of relatedness stemming from these so that depressed persons are able to restore a kind of experiential and practical openness of their existential situation.

The following quote illustrates that this may mean hard work, as to be existentially situated in a non-depressive way often has become literally unthinkable for the severely depressed. Alternative modes of being related to the world stay out of experiential and practical reach in feeling, thought, desire, embodiment and action:

“When you are depressed, the past and the future are absorbed entirely by the present moment, as in the world of a three-year-old. You cannot remember a time when you felt better, at least not clearly; and you certainly cannot imagine a future time when you will feel better. Being upset, even profoundly upset, is a temporal experience, while depression is a-temporal. Breakdowns leave you with no point of view.” (Solomon, 2001,p. 55).

The quote from Solomon illustrates how the fundamental restriction of one’s existential situation becomes transparent in terms of temporality, too. The reverberation of a non-depressive past and the anticipation of a future without having depression are fundamentally restricted, as one sticks to the present moment, which forms an isolated and disconnected experience of presence (for detailed analysis of altered temporality in psychopathology, see, e.g., cf. Bech, 1975; Ciompi, 1988; Mundt et al., 1998; Fuchs, 2001, 2005a, 2013; Habermas et al., 2008). As depression is often a long-term condition, the “depressive” evaluative patterns can become part of the habitual evaluative repertoire by means of their structural rigidity, which then shapes the *depressive habitus*^6^. With respect to the self-perpetuating evaluative dynamics by which the depressive situation enfolds as a long-term condition, a chronic disease management model for depression is needed (cf. Andrews, 2001). At least, the high co-morbidity of depressive symptoms with long-term conditions, e.g., diabetes (cf. Anderson et al., 2001), heart diseases, anxiety disorders (e.g., Paschalides et al., 2004), and physical conditions with inflammatory processes (cf. Harrison et al., 2009), point toward the relevance of perceiving the depressive situation in lights of long-term impairment. This is reflected in its conceptualization as a specific type of existential situation that challenges individuals to develop long-time oriented coping strategies (cf. e.g., McEvoy and Barnes, 2007; Smit et al., 2007; Naylor et al., 2012) to counterbalance the structural changes of evaluation by which it manifests as a harmful condition of existential narrowness.

### (DISTORTIONS OF) PRACTICAL SENSE IN DEPRESSION

It is with respect to the openness of one’s existential situation that one is able to re-evaluate and to readjust in the light of new experiences and practice of sense-making.

#### “I can(not)”

Normally, we register when things might (have) go(ne) wrong, and when particular modes of meaningful relatedness become inherently problematic. It is in virtue of *practical sense*, that we realize the possibilities and restrictions, thus the opportunities and limits for reassessment and readjustment in our life. The notion of *practical* sense reminds us of that according to which the world and self are perceived not solely in terms of an abstract “I think that,” but in terms of an “I can” as Maurice Merleau-Ponty emphasizes in his *Phenomenology of Perception* (Merleau-Ponty, 1962, Part I, Chapter 3, § 19, p. 159). Martin Heidegger (cf. Heidegger, 1962, 114, § 18) expresses the idea that the world is perceived as a place in which things appear to individuals not only as “present-at-hand” (*German: Vorhandenheit*), but is normally experienced as a place of (normative) affordances, according to which things appear to them as “ready-to-hand” (*German: Zuhandenheit*). Practical sense apparently influences not only *what* we do in a specific situation, but significantly contributes to *how* we are existentially situated in the world, i.e., how we enact in and through processes of pre-reflective and reflective evaluation, by which a particular meaningful relation between self, others and the world is instantiated (cf. Jacobs, 2013a). As such, it provides us also with modes for readjustment, for coping and adaptation by implementing a “world of possibilities.” This is also integral in Husserl’s (1960) and Merleau-Ponty’s (1962) concepts of *horizon*, where the horizontal structure of experience particularly points to how the body sets up the world, and how this is implicated in someone’s particular experience of the world. Although many alterations of practical sense cannot be labeled pathological *per se*, existential situations of the pathological type very often include significant distortions of practical sense.

#### Evaluative incoherence in depression

In psychopathological conditions, like depression, such experiences of being vitally connected to the world and others through the body, which normally is a transparent medium for evaluative self- and world-disclosure, can become severely distorted (cf. Svenaeus, 2000; Fuchs, 2005b, c) as the following quote from Solomon illustrates:

“I found everything excruciatingly difficult, and so, for example, the prospect of lifting the telephone receiver seemed to me like bench pressing four hundred pounds.” (Solomon, 2001, p. 85)

This is one way to account for a distortion of practical sense in depression. The question is, whether one can address on the bodily corporal level a fragmentation of the experiential unity in evaluative self- and world-disclosing processes with this example: I believe that the experience of “difficulty” in this example, indeed, reflects a type of experiencing evaluative incoherence. The appraisal is not solely provided by reflective assessment, but given in virtue of pre-reflective evaluative appraisals. Incoherence does play a role, insofar as one perceives the actual bodily corporal condition (“I cannot”) always against the backdrop of the condition (the bodily corporal “I can”) prior to the onset of one’s depression. The incorporated sense of being able to behave and to act as one normally does, becomes fragile, i.e., corporal-bodily possibilities cannot be actualized in such specific situations of being unable to even lift the telephone receiver.

“Evaluative incoherence” also captures more severe cases of estrangement, alienation, and even de-realization/depersonalization-processes in depression. The experiential unity is fragmented in such moments of experiencing oneself no longer the center of one’s own perception or losing trust in these. Inasmuch as perceiving oneself, others and the world in a particular way already entails processes of evaluation, Thompson’s example of altered self-awareness accounts for this distortion of the experiential unity, too:

“I began to lose faith in my own perceptions. It was as if I were standing in front of a mirror which was gradually getting distorted; eventually, what I saw bore little relationship to reality, but the change had been so slow that I had no idea where the distortion began.” (Thompson, 1996, pp. 125–126)

Another option to specify distortions of practical sense is to point to the scenario in which the “I cannot” stems from being overwhelmed by the sheer concomitance of evaluative experience occurring in a specific situation, and/or from being torn by evaluative conflict. The more complex a situation is, the more likely it is also that conflicting evaluative content (desire to kill oneself vs. desire to take care for one’s children) is present, and sometimes needs to be additionally assessed against the backdrop of corresponding different evaluative types (a belief, that leaving the children is selfish vs. the feeling that nothing makes sense any longer). On an experiential level, these evaluations demand their structural integration into a consistent evaluative pattern that allows for evaluative coherence.

Structural inconsistencies normally can be solved, e.g., in self-reflexive processes, or simply resolve themselves due to the (phenomenal-intentional) strength of a particular evaluative type/evaluative pattern overriding the other(s), and thus, initiates the respective experiential and practical mode of meaningful relatedness. We often also stay in the particular evaluative state of ambivalence, which clearly presents a different type of meaningful relatedness as when one is stuck in the process of constant rumination and indecisiveness, as it is seen as symptomatic for depression (cf. DSM-IV-TR; American Psychiatric Association, 2000, p. 349; Watkins and Teasdale, 2001). The particular symptomatic experiences can be explained in terms of distortions in one’s self-reflexive evaluative processes. These become surface, for instance, in a depressive person’s inability to affirm or distance oneself from certain thoughts, desires, or feelings and to adopt an objectifying stance that normally would give one some time to recognize what one really thinks, feels, desires in a specific situation. Even if one associates constant rumination with such a self-reflexive stance, the ability to rank preferences, i.e., to create a hierarchy of motives, according to which that what is of importance, normally becomes clear to someone – seems impaired in depression, as the symptom of depressive indecisiveness suggests.

Being capable to decide is often perceived as a structural requirement for effective deliberation, and thus significantly contributes to experience of not being fundamentally determined and existentially exposed. Consequently, one could assume that if these structural divergences in evaluation – which also presents themselves as divergences of import – remain unresolved, not only the integrative unity in evaluative experience is put at risk (with Harry Frankfurt we can even claim a distortion of the synchronic unity of the *self*; cf. Frankfurt, 1988, 2006, p. 19), but in tendency also an agent’s autonomy. In emphasizing on such moments of structural evaluative inconsistency, and corresponding modes of incoherent evaluative experience, an alteration of practical sense in depression is addressed, in which the “I cannot” shows in a distortion of particular self-reflexive processes.

It can be objected, however, that there are many other ways to specify an “I cannot” in depression, and that these examples, moreover, show that it is misleading to tie evaluative incapacity too close to specific clinical symptoms. This can be outlined by a contrary case of depressive *decisiveness*. Imagine, for instance, the situation in which the desire to kick the ladder on which one has stood already for an hour with a rope around one’s neck, is neither outplayed by the desire to be a good mom, nor by feelings of love and sorrow for the children one would leave behind by committing suicide, and does not results in the decision to climb down the ladder to prepare breakfast for them, but leads one to “wholeheartedly” kick the ladder and hang oneself. The evaluative incoherence assumed as pressing in the situation, as such, is definitely solved. Respectively, it seems to represent rather a classic case of “I can” in depression, thus, rules out the description of evaluative incapacity in virtue of the clinical symptoms provided above, too.

To my defense, it can be assumed that effective suicide is frequently committed in phases of recovery (cf. Schweizer et al., 1988; Mittal et al., 2009), thus, when it is likely that someone has restored some psychic and physic resources, that may allow one to effectively deliberate, in contrast to those phases when one cannot even perform the simplest tasks of daily life. Moreover, one may doubt, whether this example really can account for an intact practical sense, albeit an “I can” is involved. The example shows that a depressed individual may be able to decide, but is not able to *care* for oneself, for other people, and for the things that have been close to one’s heart, at least in such ways that committing suicide remains the “unthinkable” option.

The example further illustrates that the “I cannot” generally allows not only for many ways to account for evaluative incoherence and particular types of incapacity, but to generally state evaluative incoherence and (in)capacity in depression as a matter of *degree*. It points toward the in-between of the two poles of global incapacity (e.g., absolute indecisiveness) vs. fully intact capacity (e.g., “wholeheartedly” committing suicide). I do not argue, indeed, that depressives *generally* face global evaluative incapacity, but that they apparently show a diminished *coping flexibly* with such stresses and strains on the evaluative structure by which the challenging normative aspects of complex evaluative situations become transparent.

### THE NORMATIVE DIMENSION OF THE DEPRESSIVE SITUATION

By relying on the notion of practical sense, one can further elaborate, how individuals calibrate their individual experiences of significance (*Sinn*) with those objective patterns of meaning (*Bedeutung*) that define what actually “makes sense” in specific contexts. As such, practical sense illustrate show subjective evaluative patterns for self- and world-disclosure become interrelated with the objective evaluative patterns of meaningful (social) practice. To be more precise: the *mediating function* of practical sense provides one not only with a sense for one’s own individual evaluative situation in terms of an “I can” (I have addressed elsewhere with a sense of agency cf. Jacobs, 2012), but also with an understanding of it *in relation* to those patterns of meaning generated in processes of inter-subjective practice (cf. Jacobs, 2013b). Individuals are able to recognize the respective patterns of meaning generated in and through social interaction, and, simultaneously, *register how they fit into that*. They experience how their individual meaningful practice matches or mismatches actual contextual (conventional, moral, social, etc.) requirements and standards of inter-subjective practice (e.g., what it *means* to be a good mom, and what sense it makes to commit suicide). It is with respect to practical sense that one counterbalances one’s evaluative experience and practice against the pre-descriptive (normative) sphere of meaning in the respective fields of social practice. With respect to modes of (re-)adjustment this implies that we do not only ask what we actually *can* do, but what *should* be all things considered the best way to do. Practical sense therein refers to more than what is captured with an “I can,” as particularly the *normative dimension of evaluative encounters* with the world in terms of an “I should” becomes addressed with it^7^.

One should note that meaningful relatedness does not have to automatically merge into the experience of a mere match of individual evaluation (e.g., moral judgment) with objective evaluative patterns (e.g., moral rules). There are several other modes of meaningful relatedness that rather rely on *distinction* (as Bourdieu, 1979 emphasizes) by which one can stress (habitual) evaluative divergence and difference as essential for one’s self-understanding and one’s world-orientation, too. Depressed individuals certainly experience themselves as distinct, but it would often be cynical to account for these experiences as modes of distinctive practice that normally are powerful tools for positioning oneself in the fields of social practice. Patricia Dunker reflects this in her novel *Hallucinating Foucault*:

“And that is the loneliness of seeing a different world from that of the people around you. Their lives remain remote from yours. You can see the gulf and they can’t. You live among them. They walk the earth. You walk on glass. They reassure themselves with conformity, with carefully constructed resemblances. You are masked, aware of your absolute difference.” (Dunker, 1996, p. 110).

This points to a specific subclass of normative conflict in depression, in which the imperatives of an “I should” (in terms of incorporated social, cultural, moral, etc. structures) stand in conflicting relation with particular experiences of an “I cannot” – as the case example of suicide has exemplarily shown – that forms a fundamental source of suffering. This is addressed in many prominent depression narratives (e.g., Plath, 1963, p. 102, 137; Styron, 1990, p. 38; Thompson, 1996, p. 47, 57), and described also in the following passage by Solomon:

“[…] I would sometimes start to cry again, weeping not only because of what I could not do, but because *the fact that I could not do it seemed so idiotic to me*. All over the world people were taking showers. Why, of why, could I not be one of them? And then I would reflect that those people also had families and jobs and bank accounts and passports and dinner plans and problems, real problems, cancer and hunger and the death of their children and isolating loneliness and failure; and I had so few problems by comparison, except that I couldn’t turn over again. […]Always at the back of my mind there was a voice, calm and clear, that said, *don’t be so maudlin; don’t do anything melodramatic*.[…] get dressed, and *do whatever it is that you’re supposed to do*. I heard that voice all the time, that voice like my mother’s. There was a sadness and a terrible loneliness as I contemplated what was lost.” (Solomon, 2001, pp. 52–53; emphasis added K. J.).

Depressed individuals experiences themselves not only as deprived of the resources to deal flexibly with the stresses and strains on their evaluative structure arising out of an “I cannot”; they simultaneously know – or at least, remember – that they normally could or even should have performed in a specific way that once was experienced as meaningful, as a quote from Duke and Hochman illustrates:

“Those periods when I stayed in bed, behind closed doors for all those weeks, I felt dirty, smelly. Part of me thought *I should* get up and wash myself, and then I would dismiss that because I couldn’t get up. My thoughts would vary from blaming others to wishing for the absolutely unattainable peace of mind. And also thoughts of: Why is this happening to me? *Why am I like this? I’m a terrible person*.” (Duke and Hochman, 1992, Chapter 7, paragraph 19; emphasis added K. J)

Consequently, the distortion of practical sense points to the problematic evaluative self-relation that does not allow for the experiential and practical modes of transcending the depressive situation in light of an existential openness; it rather harmfully reminds one that experiencing oneself, others and the world as meaningful points of reference, has lost most of its practical significance.

## CONCLUSION

I have examined the depressive situation as a specific type of existential situation and emphasized the procedural dynamics of the pre-reflective and reflective spheres of evaluation, narrativity as a structuring principle for the experiential unity in evaluative processes, processes of evaluative disintegration and experiences of evaluative incoherence, and how these appear as distortions of practical sense in depression. First person accounts of depression from the memoirs genre served to illustrate these contexts. These have evidenced changes of meaningful relatedness in depression in pointing to recurrent experiential patterns of evaluative incoherence and incapacity, and moreover to the normative challenges associated with these. It seems that some of the phenomena I have discussed should be given more attention in diagnostic-clinical theory and practice. Although these may not share the grounds of operationalization, and respective validity and reliability as claimed for the official criteria listed in the diagnostic manuals, they may be of diagnostic value in addition to the current criteria. It is, moreover, another issue to show how such phenomenological descriptions of evaluative experience can be emphasized more in the context of future empirical research that aims to single out the neuropsychological correlates of the complex dynamics of pre-reflective and reflective evaluative self- and world-disclosing processes, and how these can substantiate some considerations of characteristic distortions in depression that have been discussed.

Although my analysis is restricted in scope, I believe that I have provided some reasons to accept that a holistic perspective on (changes in) evaluative processes in depression may contribute to a better understanding of its characteristic modes of self- and world-disclosure.

Future research will focus on an even more detailed analysis of salient changes of meaningful relatedness in psychopathology, especially with respect to the bodily corporal dynamics as constitutive for processes of normative self- and world-disclosure. A related methodological topic is to continue relating phenomenological considerations closer to empirical research and normative considerations about such specific concepts as agency, capacity, and autonomy, which are central to the notion of existential situation.

## Conflict of Interest Statement

The author declares that the research was conducted in the absence of any commercial or financial relationships that could be construed as a potential conflict of interest.

## Footnotes

1 For a detailed discussion of the distinction of “illness” and “disease,” see Boorse (1975) and Schramme (2003).

2 Normally, self-reflection is understood as a mode of intentionality that improves one’s practice in the future through a *retrospective* analysis of action. The difference to “self-reflexivity” becomes relevant with regard to when this process of introspection exactly takes place, as self-reflexivity refers to the *ongoing evaluative process*, for instance in deliberation.

3 I have elsewhere provided an answer in terms of a synthesis-account in order to tackle some problems of classic cognitivist and component theories of *emotion* (cf. Jacobs et al., 2013). This has been the background for claiming that an adequate theory of evaluation in general – and not only theories of emotions – has to explain both, the intentionality and phenomenology of evaluation (cf. Goldie, 2000; Helm, 2001; Döring and Peacocke, 2002; Stephan and Slaby, 2011), and how these form an *integrative unity in processes of evaluative self- and world-disclosure*, in particular.

4 Inasmuch as my account focuses on experiences of fragmentation due to significant structural changes in the evaluative processes in depression, it is evident that I do not stipulate such fragmentations as the experiential norm. It has to be discussed elsewhere, whether emphasizing on the latter would require to reconsider narrativity to be a necessary structuring principle, as it is, for instance, suggested by (1996, p. 174), who emphasizes on “discordance concordance” that likewise accounts for a synthesis of heterogeneous aspects of one’s evaluative situation in virtue of narrativity as a (necessary) structuring principle.

5 A bias is “a proclivity to take one direction over another which under same conditions will lead to accuracy or realism, but under other conditions will lead to inaccuracy”; while “distortion” implies something invariably wrong (cf. Power, 1991; Power and Dalgleish, 2008, p. 247).

6 With Bourdieu’s theory, the bodily disposition and specific gestures, postures and body-language of depressive persons can be systematized and decoded as reflecting the historicity and actuality of a depressed individual’s whole bio-psycho-social status. These aspects of “depressive habitus” exemplarily have been detailed by the German psychiatrist Hubertus Tellenbach, who addresses the habitual dimension of depression – inclusive its characteristic *hexis* – with his famous “*Typus Melancholicus*” (cf. Tellenbach, 1961; see also Jaspers, 1913, 141–144; § 5; Fuchs, 2006).

7 The notion of practical sense therein contributes to an understanding of social, particularly: *moral* practice. A lack of moral sense (moral incapacity) can be addressed as a subtype of distortion of practical sense to experience oneself as meaningfully (morally) related to others (cf. Jacobs, 2012, 2013a).
